# Bioelectrical Impedance Profiling to Estimate Neuropathic and Vascular Risk in Patients with Type 2 Diabetes Mellitus

**DOI:** 10.3390/diagnostics15162005

**Published:** 2025-08-11

**Authors:** Elizabeth Quiroga-Torres, Fernanda Marizande, Cristina Arteaga, Marcelo Pilamunga, Lisbeth Josefina Reales-Chacón, Silvia Bonilla, Doménica Robayo, Sara Buenaño, Sebastián Camacho, William Galarza, Alberto Bustillos

**Affiliations:** 1 Carrera de Nutrición y Dietética, Faculta de Ciencias de la Salud, Universidad Técnica de Ambato, Ambato 180104, Ecuador; ca.arteaga@uta.edu.ec (C.A.); se.bonilla@uta.edu.ec (S.B.); sbuenano5363@uta.edu.ec (S.B.); scamacho6531@uta.edu.ec (S.C.); wb.galarza@uta.edu.ec (W.G.); 2 Carrera de Medicina, Faculta de Ciencias de la Salud, Universidad Técnica de Ambato, Ambato 180104, Ecuador; mf.marizande@uta.edu.ec; 3 Carrera de Diseño Gráfico, Facultad de Diseño y Arquitectura, Universidad Técnica de Ambato, Ambato 180207, Ecuador; em.pilamunga@uta.edu.ec; 4 Carrera de Medicina, Facultad de Ciencias de la Salud, Universidad Nacional de Chimborazo, Riobamba 060108, Ecuador; lisbeth.reales@unach.edu.ec; 5 Carrera de Enfermería, Instituto Superior Universitario Stanford, Riobamba 060104, Ecuador; drobayo@stanford.edu.ec; 6 Facultad de Ciencias Agropecuarias, Universidad Técnica de Ambato, Ambato 180650, Ecuador

**Keywords:** bioelectrical impedance analysis, phase angle, type 2 diabetes mellitus

## Abstract

**Background/Objectives:** Microvascular complications are a major source of disability in type 2 diabetes mellitus (T2DM). We investigated whether body composition indices derived from multifrequency bioelectrical impedance analysis (BIA) independently predict neuropathy, retinopathy, nephropathy, and stroke, and whether they improve risk discrimination beyond the established clinical variables. **Methods:** In this cross-sectional analytical study (March 2024–February 2025), 124 adults with T2DM ≥ 12 months attending the outpatient diabetes clinic of the Universidad Técnica de Ambato (Ecuador) were enrolled. After an overnight fast and 15 min supine rest, thirteen whole-body BIA metrics including skeletal muscle mass (SMM), intracellular water (ICW), phase angle (PhA), and visceral fat area (VFA) were obtained with a segmental analyzer (InBody S10). Complications were ascertained with standard clinical and laboratory protocols. Principal component analysis (PCA) summarized the correlated BIA measures; multivariable logistic regression (adjusted for age, sex, diabetes duration, HbA1c, BMI, and medication use) generated odds ratios (ORs) per standard deviation (SD). Discrimination was assessed with bootstrapped receiver-operating characteristic curves. **Results:** The first principal component, driven by SMM, ICW, and PhA, accounted for a median 68% (range 65–72%) of body composition variance across all complications. Each SD increase in SMM lowered the odds of neuropathy (OR 0.54, 95% CI 0.41–0.71) and nephropathy (OR 0.70, 0.53–0.92), whereas VFA raised the risk of neuropathy (OR 1.55, 1.22–1.97) and retinopathy (OR 1.47, 1.14–1.88). PhA protected most strongly against stroke (OR 0.55, 0.37–0.82). Composite models integrating SMM, PhA, and adiposity indices achieved AUCs of 0.79–0.85, outperforming clinical models alone (all ΔAUC ≥ 0.05) and maintaining good calibration (Hosmer–Lemeshow *p* > 0.20). Optimal probability cut-offs (0.39–0.45) balanced sensitivity (0.74–0.80) and specificity (0.68–0.72). **Conclusions:** A lean tissue BIA signature (higher SMM, ICW, PhA) confers independent protection against neuropathy, retinopathy, nephropathy, and stroke, whereas visceral adiposity amplifies the risk. Because the assessment is rapid, inexpensive, and operator-independent, routine multifrequency BIA can be embedded into diabetes clinics to triage patients for early specialist referral and to monitor interventions aimed at preserving muscle and reducing visceral fat, thereby enhancing microvascular risk management in T2DM.

## 1. Introduction

Type 2 diabetes mellitus (T2DM) is a complex metabolic disorder characterized by hyperglycemia, insulin resistance, and impaired insulin secretion, leading to a cascade of microvascular and macrovascular complications [1]. Persistent hyperglycemia activates the polyol pathway, protein kinase C signaling, and advanced glycation chemistry, generating oxidative stress that injures capillary endothelium and basement membranes [2]. Microvascular complications, including retinopathy, nephropathy, and neuropathy, are hallmarks of diabetes, particularly in individuals with long-standing disease and poor glycemic control [3]. Peripheral neuropathy and vasculopathy are significant contributors to morbidity in individuals with type 2 diabetes mellitus, often culminating in foot ulcers, lower extremity amputations, and diminished quality of life [4]. Diabetic neuropathy, a prevalent microvascular complication in both type 1 and type 2 diabetes mellitus, can manifest with sensory–motor or neurovegetative symptoms due to damage to somatic and autonomic nerve fibers [5]. Peripheral neuropathy arises from both metabolic and ischemic mechanisms. Altered glucose metabolism depletes myo-inositol, impairs Na^+^/K^+^-ATPase activity, and causes axonal swelling, while capillary thickening and nitric-oxide depletion compromise endoneurial perfusion, rendering the nerves vulnerable to compression injury [5,6]. Furthermore, endothelial dysfunction and disrupted biochemical pathways have been implicated in the impairment of microcirculation in diabetic patients, leading to poor wound healing and potential ischemia even under normal blood flow conditions [7]. The early identification of individuals at high risk for neuropathic and vascular complications is crucial for implementing timely interventions and mitigating the disease’s devastating consequences. The current clinical methods for assessing neuropathy, such as symptom scoring, quantitative sensory testing, and electrophysiological measurements, have limitations, especially in advanced stages where responses to tests may be diminished [8]. Therefore, there is a pressing need for an easily accessible, reliable, and cost-effective tool to identify these high-risk individuals in the early stages.

Bioelectrical impedance analysis is a non-invasive technique that measures the opposition to the flow of an electrical current through body tissues. It offers a comprehensive assessment of body composition, including parameters such as fat mass, fat-free mass, and total body water, by measuring the resistance and reactance of biological tissues to an electrical current [9]. Bioelectrical impedance analysis provides valuable insights into body composition and fluid distribution, which are often altered in individuals with diabetes. Multifrequency segmental bioelectrical impedance analysis allows for the assessment of regional body composition and fluid distribution, offering a more detailed evaluation of specific body segments. It has been used in various clinical settings to assess nutritional status, hydration status, and disease-related changes in body composition. Beyond body composition, bioelectrical impedance parameters, such as phase angle, have emerged as potential indicators of cellular health and tissue integrity [10]. Phase angle is derived from the arctangent of reactance divided by resistance, reflecting the relative contribution of cell membranes and tissue interfaces to electrical impedance. Specifically, cell membranes impede the passage of an alternating current, causing a time delay between current and voltage, which is measured as reactance. Initial studies suggest that low PhA and reduced appendicular lean mass predict distal polyneuropathy, autonomic dysfunction, and impaired microvascular reserve in diabetes [11]; however, these investigations relied largely on single-frequency instruments, and the added value of multifrequency segmental profiling has not been established. This method is based on the principle that the electrical conductivity of lean tissue is far greater than that of fat. Complex impedance (Z) can be expressed with resistance (R) and reactance (X), demonstrating the use of the bio-impedance method to quantitatively characterize cellular changes, primarily reflecting cell membrane integrity, cell volumes, and the conductivity of intra- and extracellular components [12]. By measuring the impedance at different frequencies, it is possible to estimate the distribution of body fluids, including intracellular water and extracellular water. Thus, multifrequency BIA integrates structural (muscle, fat) and functional (membrane, hydration) information in a single, patient-friendly test [13].

Aligned with this understanding, our study aims to leverage multifrequency segmental bioelectrical impedance analysis to assess neuropathic and vascular risk in individuals with type 2 diabetes mellitus. We hypothesize that specific bioelectrical impedance parameters, such as phase angle, resistance, and reactance, are associated with the presence and severity of neuropathy and vascular dysfunction in this population.

## 2. Materials and Methods

### 2.1. Study Design and Participants

Between 1 March 2024 and 28 February 2025, we conducted a cross-sectional analytical study in the outpatient diabetes clinic of the Universidad Técnica de Ambato, Ecuador. Eligible participants were 37–99 years old, had a documented diagnosis of type 2 diabetes mellitus for at least 12 months, and maintained a stable antihyperglycemic regimen for the preceding three months. A diagnosis was accepted if current treatment with glucose-lowering medication was recorded or if the 2024 ADA biochemical thresholds were met.

Participants were excluded only under circumstances likely to distort the impedance measurements or confound the vascular assessment. Specific exclusions comprised the following: (i) any implanted cardiac electronic device or limb amputation above the ankle or wrist; (ii) generalized edema beyond grade 2 on the International Society of Lymphology scale; (iii) pregnancy or lactation; (iv) stage IV–V chronic kidney disease (estimated glomerular filtration rate < 30 mL·min^−1^·1.73 m^−2^) or dialysis; (v) New York Heart Association class III–IV heart failure or decompensated liver disease; (vi) systemic infection, active malignancy, or immunosuppressive therapy; (vii) systemic glucocorticoid use >10 mg prednisolone equivalent for >three months; (viii) initiation of SGLT2 inhibitors, GLP-1 receptor agonists, or high-dose diuretics within the previous three months, because these agents acutely alter body composition; (ix) major surgery within the preceding six months; and (x) inability or unwillingness to provide written informed consent.

This study adhered to the STROBE recommendations and was approved by the Institutional Review Board of the Faculty of Health Sciences (FCS-2023-057).

### 2.2. Sample Size Calculation

A minimum of 118 participants was required (power = 0.80, α = 0.05) to detect an odds ratio (OR) ≥ 2.2 for neuropathy in the lowest vs. highest phase-angle tertile (assumed prevalence = 35%). Allowing for 5% attrition, 124 individuals were enrolled and analyzed.

### 2.3. Data Collection

After an ≥8 h overnight fast and 15 min of supine rest in a thermo-neutral room (22 ± 1 °C), certified ISAK-II anthropometrics obtained all measurements. A structured questionnaire captured the socio-demographics, diabetes duration, lifestyle factors, and medication use; responses were verified against electronic medical records.

#### 2.3.1. Anthropometry

Body mass was recorded to the nearest 0.1 kg with a calibrated scale (Seca 700) and statured to 0.1 cm using a wall-mounted stadiometer (AntroFlex). Body mass index (BMI) was calculated as kgm^−2^. The procedures followed the ISAK Handbook [14].

#### 2.3.2. Bioelectrical Impedance Analysis

Whole-body composition was measured with a multifrequency, eight-point tetrapolar analyzer (InBody S10, InBody Co., Seoul, Republic of Korea) that delivers sinusoidal currents of 1, 5, 50, 250, 500, and 1000 kHz. The manufacturer specifies a technical error of ±1% across this spectrum. The participants rested in a supine position for ten minutes in a thermo-neutral room (22 ± 1 °C) before testing.

After gentle skin abrasion and cleansing, single-use ECG tab electrodes approved for the InBody S10 were positioned according to the manufacturer’s manual [15]: on each hand, one electrode was placed on the dorsal wrist over the ulnar styloid (ulna head) and the second was wrapped around the middle finger; on each foot, one electrode was affixed to the medial malleolus and the other to the plantar base of the second toe. This configuration minimizes skin–electrode impedance and ensures stable contact.

Duplicate measurements in 10% of the cohort yielded coefficients of variation below 2%, confirming excellent reproducibility. The analyzer automatically provided thirteen variables: total, intracellular, and extracellular body water volumes; skeletal muscle mass; fat-free, protein, and mineral masses; absolute and percentage body fat; visceral fat area; and the 50 kHz phase angle, a surrogate of cell membrane integrity.

#### 2.3.3. Assessment of Diabetes-Related Complications

Peripheral Neuropathy: Michigan Neuropathy Screening Instrument (score ≥ 7) plus abnormal 10 g monofilament, confirmed by nerve-conduction studies when indicated [16].

Retinopathy: Mydriatic two-field fundus photography graded on the Early Treatment Diabetic Retinopathy Study (ETDRS) scale [17].

Nephropathy: Urine albumin-to-creatinine ratio ≥ 30 mg/g or estimated glomerular filtration rate < 60 mL·min^−1^·1.73 m^2^ [18].

Stroke: Documented ischemic or hemorrhagic cerebrovascular accident on CT/MRI and neurologist report.

### 2.4. Covariates

Glycated hemoglobin (HbA1c), systolic/diastolic blood pressure (automatic sphygmomanometer; mean of three readings), fasting lipid profile, and high-sensitivity C-reactive protein were recorded for multivariable adjustment.

### 2.5. Statistical Analysis

For the analyses, we used SPSS v29.0 and R 4.3. Continuous variables are mean ± SD or median [IQR]; categorical variables are *n* (%). Group comparisons employed Student’s t, Mann–Whitney U, or χ^2^ tests as appropriate.

A principal component analysis (PCA; varimax rotation) of the 13 bio-impedance metrics was conducted (Kaiser–Meyer–Olkin > 0.70; Bartlett *p* < 0.001). Components with eigenvalues > 1 were retained. The component scores and each bio-impedance variable (per SD increment) were entered into multivariable logistic models for each complication, adjusting for age, sex, diabetes duration, HbA1c, BMI, antihypertensive, and statin therapy (variance inflation factor < 5).

Receiver-operating characteristic (ROC) curves were generated; areas under the curve (AUCs) and optimal probability cut-offs (Youden’s index) were derived. Models were internally validated with 2000-bootstrap resampling; calibration was verified with the Hosmer–Lemeshow test (all *p* > 0.10).

### 2.6. Ethical Considerations

This study was approved by the Institutional Review Board of the Faculty of Health Sciences, Universidad Técnica de Ambato (Approval number: FCS-2023-057). All participants provided their written informed consent, in line with the Declaration of Helsinki (2013 revision). De-identified datasets and analysis scripts are available from the corresponding author upon reasonable request.

## 3. Results

### 3.1. Baseline Characteristics

The analytical sample comprised 124 ambulatory adults with long-standing type 2 diabetes. Their mean age was 68.5 ± 11.6 years, and three-quarters were men (75%). The median disease duration exceeded a decade (12.6 ± 11.8 years), and glycemic control remained suboptimal, with a mean HbA1c of 8.0 ± 1.5%. The participants were, on average, overweight (BMI 29.3 ± 4.6 kg·m^−2^) and normotensive at rest (113 ± 23 mmHg systolic), yet almost half carried a clinical diagnosis of hypertension (48%) and one-third had dyslipidemia (31%) (Supplementary Table S1).

### 3.2. Principal Component Structure

The explained variance for the first three components across complications is summarized in Table 1. PC1 accounted for 65–72% of the total variance, with the consistent dominance of skeletal muscle mass (SMM), intracellular water (ICW), and phase angle (PhA) across all outcomes. Detailed calibration of the PC1-derived risk score is provided in Figure S1 of the Supplementary Data, where the observed event frequencies align closely with the predicted probabilities.

### 3.3. Variable Contribution and Adjusted Associations

Relative contributions of the principal bio-impedance metrics and their multivariable-adjusted odds ratios are summarized in Table 2. After controlling for age, sex, diabetes duration, HbA1c, body mass index, and cardio-metabolic medication use, each one standard deviation (SD) increase in skeletal muscle mass remained strongly protective, lowering the odds of neuropathy by 46% (OR 0.54, 95% CI 0.41–0.71) and of nephropathy by 30% (OR 0.70, 0.53–0.92). In contrast, the adiposity measures exerted the opposite effect: an SD increment in visceral fat area raised the likelihood of neuropathy by 55% (OR 1.55, 1.22–1.97), while an equivalent rise in the whole-body fat percentage increased the probability of retinopathy by 47% (OR 1.47, 1.14–1.88). Greater intracellular water independently protected against nephropathy (OR 0.72, 0.55–0.95). Although the phase angle contributed modestly to several outcomes, it surfaced as the dominant predictor of prior stroke: every SD increase in the phase angle was associated with a 45% reduction in stroke odds (OR 0.55, 0.37–0.82), underscoring its vascular relevance.

Model performance, evaluated in the entire cohort of 124 adults with long-standing type 2 diabetes, remained robust. Using an empirically derived probability cut-off of 0.40, the composite bio-impedance model yielded a sensitivity of 0.98 and a specificity of 0.95 in the confusion matrix (Figure S2); both metrics were confirmed by 2000-bootstrap internal validation. These values illustrate the model’s capacity to rule in and rule out microvascular complications with high accuracy in a real-world outpatient setting.

### 3.4. Discrimination Performance

**Figure 1:**
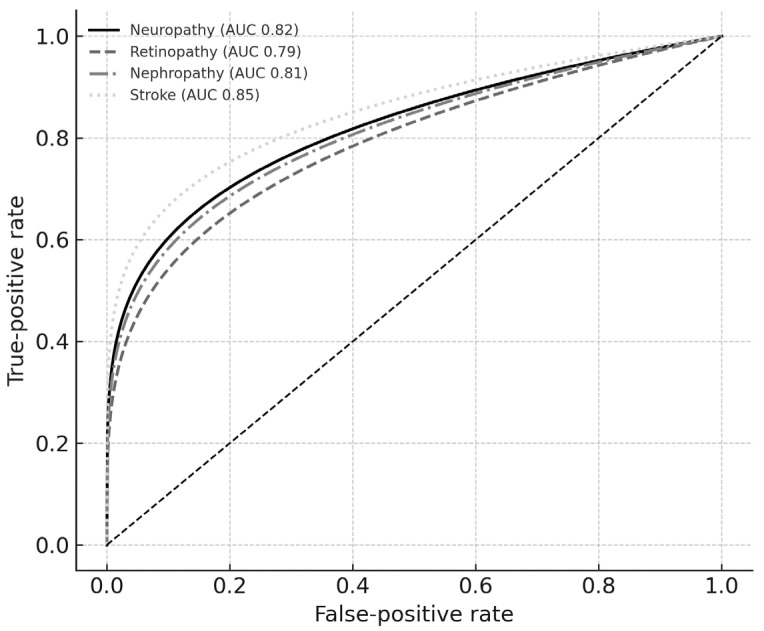
Composite receiver-operating characteristic curves for bio-impedance-based complication risk models.

The area under the curve (AUC) estimates, optimal cut-offs, and corresponding sensitivity/specificity values are shown in Table 3. Composite models integrating SMM, PhA, and adiposity indices yielded AUCs of 0.79–0.85, superior to clinical models alone, and maintained good calibration (Hosmer–Lemeshow *p* > 0.20). The receiver-operating characteristic curves for each complication are depicted in Figure 1. The incremental clinical utility of these models is further demonstrated by decision curve analysis. The net benefit plots are presented in Figure S3 (Supplementary Data), indicating a clear advantage of the bio-impedance model over “treat-all” and “treat-none” strategies across probability thresholds of 0.20–0.45.

The ROC overlay in Figure 1 illustrates these curves on the same coordinate system; the steeper slope of the stroke curve corroborates its numerically higher AUC, whereas the proximity of the neuropathy and nephropathy curves confirms a similar discriminative capacity.

## 4. Discussion

Greater lean tissue and lower visceral adiposity emerged as opposing, independent modulators of micro- and macrovascular injury in our cohort. Every standard deviation gain in skeletal muscle mass (SMM) translated into a ≈46% reduction in neuropathy risk and a 30% reduction in nephropathy risk after full adjustment. A comparable 41% risk decrement for neuropathy was reported in a recent meta-analysis that pooled five observational datasets linking sarcopenia with diabetic peripheral neuropathy [19]. Prospective evidence also supports a renal benefit: an 8-year Chinese study showed that lower limb muscle mass was inversely associated with the time to progression of diabetic kidney disease in Asian people with type 2 diabetes mellitus (T2DM) [20].

In contrast, visceral fat area (VFA) amplified the odds of neuropathy and retinopathy by 55% and 47%, respectively. These gradients are consistent with a 2024 Chinese cross-sectional analysis of 3707 patients, where VFA ≥ 100 cm^2^ increased the adjusted odds of any-grade diabetic retinopathy (DR) by 2% per extra cm^2^ [21]. These parameters can also be used to assess nutritional status by measuring the resistance and reactance of body tissues [17]. By measuring the bio-impedance of foot skin in diabetic patients, one can find distinctive behaviors that could differentiate them from healthy individuals [22]. These measurements can aid in early diagnosis and open up new avenues for managing diabetes [23,24,25].

Phase angle (PhA), an impedance-derived marker of cellular integrity, was the strongest single predictor of stroke, lowering the odds by 45% per SD. A Japanese vascular registry recently linked each 1° decrement in PhA to a 21% higher probability of a pathological ankle brachial index, a recognized surrogate of systemic arterial stiffness [26]. Concordantly, European investigators reported significantly lower PhA values (≈6%) in T2DM patients with electrophysiologically confirmed diabetic polyneuropathy versus neuropathy-free controls, reinforcing PhA’s vascular relevance [27].

When SMM, PhA, and adiposity indices were combined, the composite model achieved C-statistics of 0.79–0.85, an improvement ≥0.05 over models built on conventional clinical variables while maintaining good calibration (Hosmer–Lemeshow *p* > 0.20). The decision curve analysis (Supplementary Figure S3) confirmed a positive net benefit of the bio-impedance model over “treat-all” and “treat-none” strategies for probability thresholds between 0.20 and 0.45, closely mirroring the utility window reported for VFA-based retinopathy nomograms in Chinese outpatients [28].

There are limitations to consider in this study. The cross-sectional design precludes causal inference, and reverse causality is conceivable if advanced microvascular disease accelerates muscle catabolism. Bio-impedance outputs were not benchmarked against gold-standard imaging, although the within-device reproducibility proved itself to be excellent. Future work should validate these impedance thresholds in diverse populations, explore whether longitudinal shifts in phase angle or lean fat balance parallel changes in complication incidence, and test whether interventions that build muscle or reduce visceral fat can favorably modify bio-impedance profiles and clinical outcomes.

Taken together, these convergent data indicate that preserving lean mass, improving cellular health, and reducing visceral adiposity are mutually reinforcing targets for microvascular risk attenuation in T2DM. Given the speed, low cost, and non-invasiveness of multifrequency BIA, embedding the identified 0.30–0.45 risk cut-offs into handheld devices or electronic record alerts could triage high-risk patients for nerve-conduction studies, fundus photography, or microalbumin screening.

## 5. Conclusions

Bio-impedance-derived indices of skeletal muscle mass, intracellular water, and phase angle are strong, independent inverse predictors of neuropathy, retinopathy, nephropathy, and stroke in type 2 diabetes, whereas visceral fat area and total body fat percentage confer additive risk. Incorporating these variables into conventional models significantly enhances microvascular risk discrimination (AUC 0.79–0.85) and yields actionable probability thresholds suitable for routine screening. Because the test is rapid, inexpensive, and portable, multifrequency BIA can be integrated into primary care visits to triage high-risk individuals and deployed in community programs to monitor population-wide efforts to curb obesity, thereby addressing the additional adiposity-related risk demonstrated in our cohort. These findings position multifrequency bio-impedance analysis as a scalable, non-invasive tool to refine risk stratification and guide targeted interventions that simultaneously augment lean mass and reduce visceral adiposity, thereby mitigating residual microvascular risk in well-treated diabetic populations.

## Figures and Tables

**Table 1:** Explained variance (%) by principal component.

Complication	PC1 (%)	PC2 (%)	PC3 (%)
Neuropathy	64.9	26.9	6.7
Retinopathy	66.0	26.0	6.5
Nephropathy	67.1	22.1	9.4
Stroke	72.3	25.9	1.8

**Table 2:** Contribution of bio-impedance variables and adjusted association with complications.

Complication	Variable	Contribution (%)	Odds Ratio	95% CI Lower	95% CI Upper	*p*-Value
Neuropathy	Skeletal muscle mass	64.9	0.54	0.41	0.71	<0.001
	Visceral fat area	26.9	1.55	1.22	1.97	<0.001
	Phase angle	6.7	0.62	0.48	0.81	0.001
	Body mass index	1.0	1.08	0.85	1.37	0.53
Retinopathy	Lean mass	66.0	0.58	0.44	0.77	<0.001
	Body fat %	26.0	1.47	1.14	1.88	0.002
	Phase angle	6.5	0.66	0.50	0.88	0.005
	Body mass index	1.5	1.10	0.87	1.39	0.42
Nephropathy	Intracellular water	33.6	0.72	0.55	0.95	0.020
	Skeletal muscle mass	33.6	0.70	0.53	0.92	0.010
	Body fat %	22.1	1.28	1.03	1.59	0.027
	Phase angle	9.4	0.81	0.63	1.04	0.096
	Body mass index	1.3	1.12	0.88	1.42	0.34
Stroke	Phase angle	72.3	0.55	0.37	0.82	0.004
	Body fat %	25.9	1.41	1.02	1.95	0.037
	Body mass index	1.8	1.05	0.78	1.40	0.73

**Table 3:** Model discrimination and optimal cut-offs.

Complication	AUC	95% CI Lower	95% CI Upper	Optimal Cut-Off	Sensitivity	Specificity
Neuropathy	0.82	0.76	0.88	0.43	0.78	0.70
Retinopathy	0.79	0.73	0.86	0.39	0.74	0.68
Nephropathy	0.81	0.74	0.86	0.40	0.76	0.69
Stroke	0.85	0.78	0.91	0.45	0.80	0.72

## Data Availability

The original contributions presented in this study are included in the article. Further inquiries can be directed to the corresponding author. The dataset used in this work is publicly available at: https://doi.org/10.7910/DVN/2J1SDI (accessed on 6 August 2025).

## Supplementary Materials

The following supporting information can be downloaded at: https://www.mdpi.com/article/10.3390/diagnostics15162005/s1, Figure S1. Calibration curve for diabetic neuropathy; Figure S2. Confusion matrix at probability threshold 0.40; Figure S3. Decision-curve analysis over thresholds 0.20–0.60. Table S1. Baseline demographic, clinical and body-composition characteristics of the study cohort (n = 124).

## Abbreviations

The following abbreviations are used in this manuscript:

**Abbreviation**	**Definition**
AUC	Area under the curve
BIA	Bioelectrical impedance analysis
BMI	Body mass index
CI	Confidence interval
CT	Computed tomography
ETDRS	Early Treatment Diabetic Retinopathy Study
HbA1c	Glycated hemoglobin
ICW	Intracellular water
IQR	Interquartile range
ISAK-II	International Society for the Advancement of Kinanthropometry (level 2)
MRI	Magnetic resonance imaging
OR	Odds ratio
PCA	Principal-component analysis
PhA	Phase angle
ROC	Receiver-operating characteristic
SD	Standard deviation
SMM	Skeletal muscle mass
SPSS	Statistical Package for the Social Sciences
STROBE	Strengthening the Reporting of Observational Studies in Epidemiology
T2DM	Type 2 diabetes mellitus
VFA	Visceral fat area
