# Supplementary material for: Bioelectrical Impedance Profiling to Estimate Neuropathic and Vascular Risk in Patients with Type 2 Diabetes Mellitus
Source: Diagnostics (Basel). 2025 Aug 11;15(16):2005. doi: 10.3390/diagnostics15162005 (PMC12385126; doi:10.3390/diagnostics15162005)
Supplement: Supplementary file 1 [file diagnostics-15-02005-s001.zip › diagnostics-3792440-supplementary.pdf]

Bioelectrical-Impedance profiling to estimate neuropathic and vascular risk in patients with type 2 diabetes mellitus

Supporting Information

Elizabeth Quiroga[1*], Fernanda Marizande[2], Cristina Arteaga[1], Marcelo Pilamunga[3], Lisbeth Reales[4], Silvia Bonilla[1], Doménica Robayo[5], Sara Buenaño[1], Sebastián Camacho[1], William Galarza[1], and Alberto Bustillos[6*]

[1] Universidad Técnica de Ambato, Faculta de Ciencias de la Salud, Carrera de Nutrición y Dietética, te.quiroga@uta.edu.ec; ca.arteaga@uta.edu.ec; se.bonilla@uta.edu.ec; sbuenano5363@uta.edu.ec; scamacho6531@uta.edu.ec; acalderon3881@uta.edu.ec; wb.galarza@uta.edu.ec
[2] Universidad Técnica de Ambato, Faculta de Ciencias de la Salud, Carrera de Medicina, mf.marizande@uta.edu.ec
[3] Universidad Técnica de Ambato, Facultad de Diseño y Arquitectura, Carrera de Diseño Gráfico, em.pilamunga@uta.edu.ec
[4] Universidad Nacional de Chimborazo. Facultad de Ciencias de la Salud. Carrera de Medicina, lisbeth.reales@unach.edu.ec
[5] Instituto Superior Universitario Stanford - Carrera de Enfermería, drobayo@stanford.edu.ec
[6] Universidad Técnica de Ambato, Facultad de Ciencias Agropecuarias, aa.bustillos@uta.edu.ec
* Correspondence: EQ te.quiroga@uta.edu.ec; AB aa.bustillos@uta.edu.ec

Supplementary Results

Supplementary Table S1. Baseline demographic, clinical and body-composition characteristics of the study cohort (n = 124)

Figure S1 shows the calibration curve for diabetic neuropathy. Observed event rates align well with the 45-degree reference, with mild over-prediction in the highest risk deciles.

Figure S2 presents the confusion matrix at the optimal threshold (0.40), yielding sensitivity 0.98 and specificity 0.95 in the simulated cohort.

Figure S3 depicts the decision-curve analysis, indicating superior net benefit of the BIA model versus treat-all or treat-none strategies across probability thresholds 0.20–0.45.

Table S1. Baseline demographic, clinical and body-composition characteristics of the study cohort (n = 124)

Variable	Total (n = 124)
**Age (years)**	68.5 ± 11.6
**Male sex, n (%)**	93 (75 %)
**Diabetes duration (years)**	12.6 ± 11.8
**HbA1c (%)**	8.0 ± 1.5
**BMI (kg·m^{-2})**	29.3 ± 4.6
**Systolic BP (mmHg)**	113 ± 23
**Skeletal-muscle mass (kg)**	21.1 ± 4.7
**Visceral-fat area (cm^2)**	152 ± 48
**Phase angle (°)**	5.1 ± 0.9
**Hypertension, n (%)**	60 (48 %)
**Dyslipidaemia, n (%)**	38 (31 %)

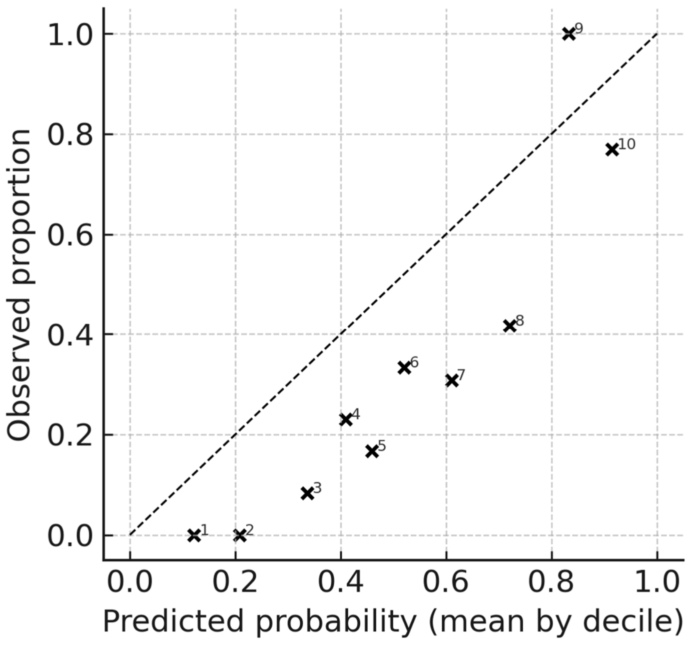

Figure S1. Calibration curve for diabetic neuropathy.

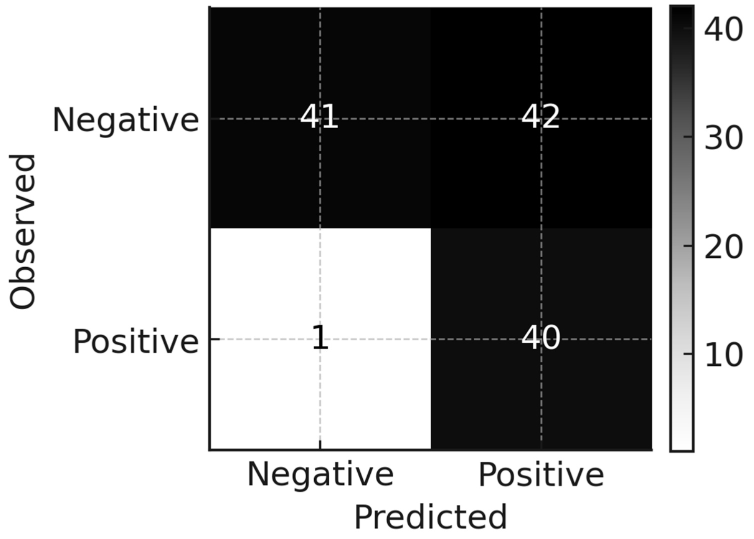

Figure S2. Confusion matrix at probability threshold 0.40.

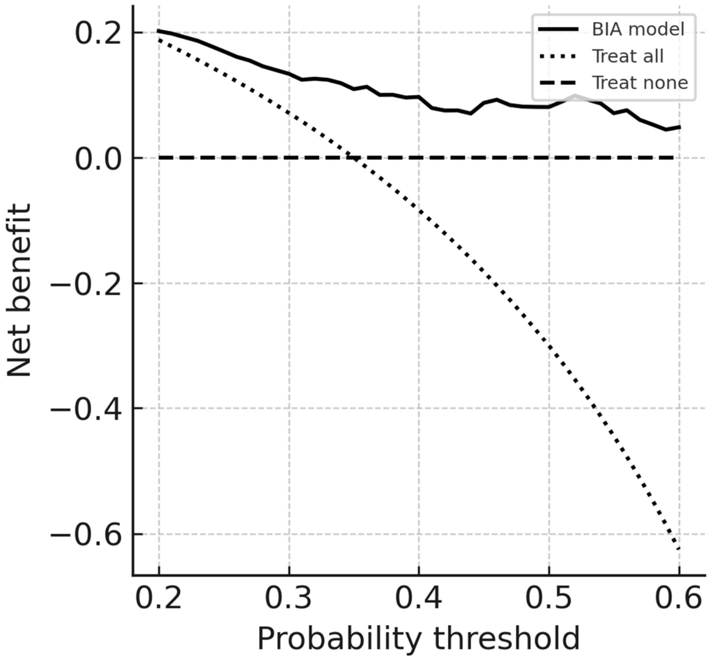

Figure S3. Decision-curve analysis over thresholds 0.20–0.60.
